# Supplementary material for: SANTO: a coarse-to-fine alignment and stitching method for spatial omics
Source: Nat Commun. 2024 Jul 18;15:6048. doi: 10.1038/s41467-024-50308-x (PMC11258319; doi:10.1038/s41467-024-50308-x)
Supplement: Supplementary file 1 — Supplementary Information [file 41467_2024_50308_MOESM1_ESM.pdf]

SANTO: a coarse-to-fine alignment and stitching method for spatial omics

Haoyang Li[1,2,†], Yingxin Lin[3,†], Wenjia He[1,2], Wenkai Han[1,2], Xiaopeng Xu[1,2], Chencheng Xu[1,2], Elva Gao[4], Hongyu Zhao[3,*], Xin Gao[1,2,*]

[1]Computational Bioscience Research Center, King Abdullah University of Science and Technology (KAUST), Thuwal, Saudi Arabia
[2]Computer, Electrical and Mathematical Sciences and Engineering Division, King Abdullah University of Science and Technology (KAUST), Thuwal, Saudi Arabia
[3]Department of Biostatistics, Yale University, New Haven, CT, USA
[4]The KAUST school, King Abdullah University of Science and Technology (KAUST), Thuwal 23955-6900, Saudi Arabia

*Correspondence should be addressed to X.G. and H.Z.
(Email: xin.gao@kaust.edu.sa and hongyu.zhao@yale.edu)
†These authors contributed equally to this work.

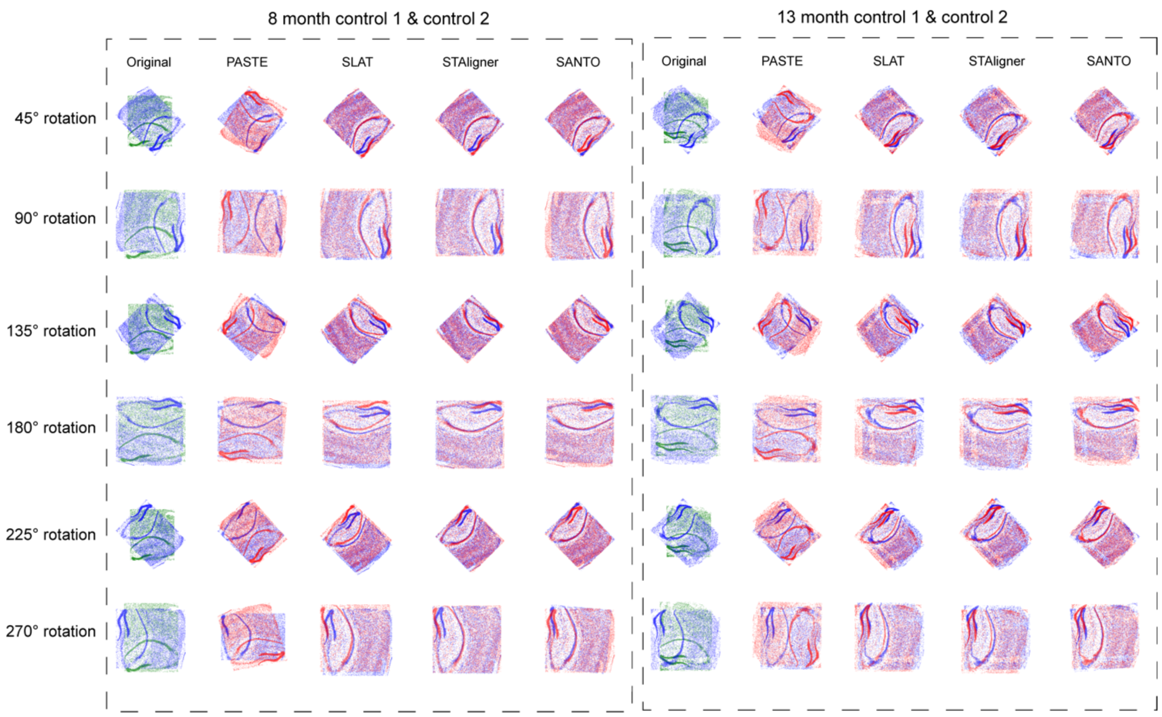

Supplementary Figure 1. The visualization results of original positions, PASTE, SLAT, STAligner and SANTO under 6 different rotations from 8- and 13-month samples.

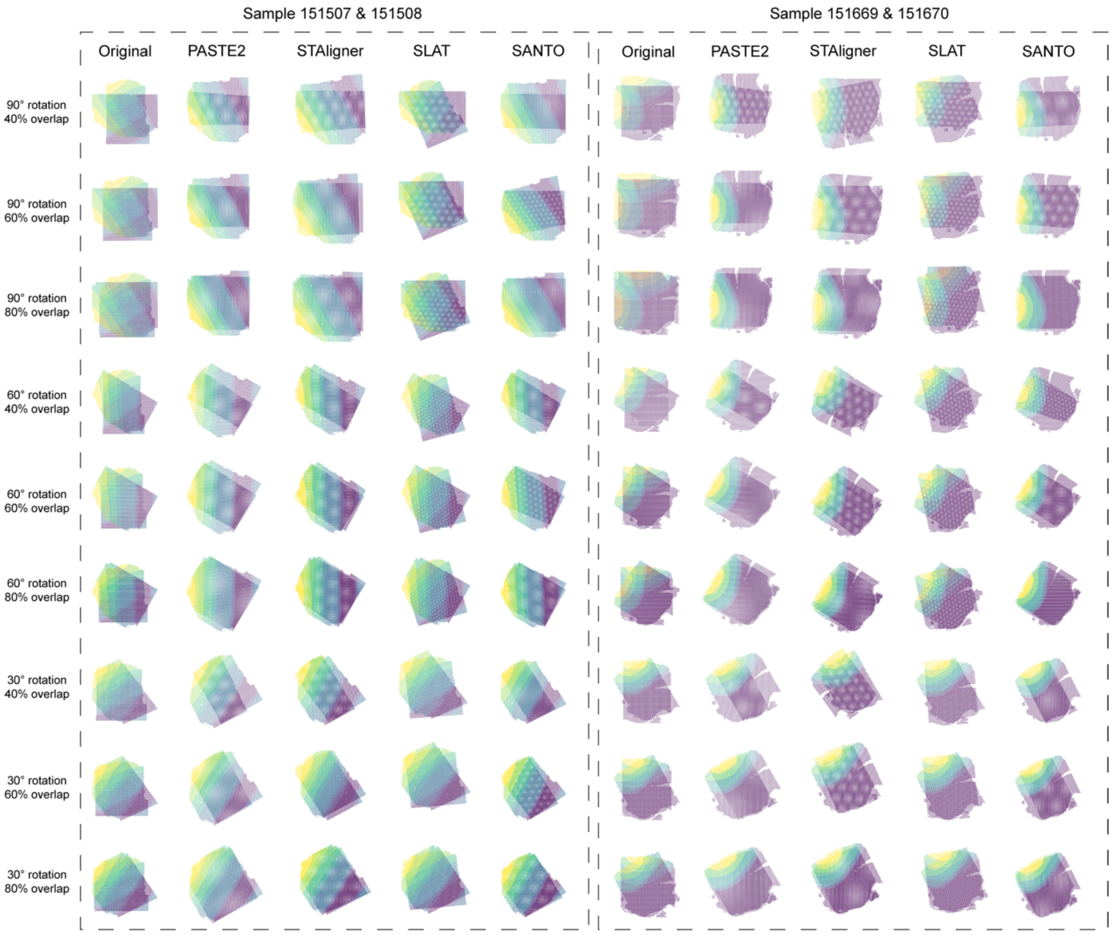

Supplementary Figure 2. The visualization results of original positions, PASTE2, SLAT, STAligner and SANTO under nine different rotations and percentages of overlap regions from two samples of DLPFC dataset.

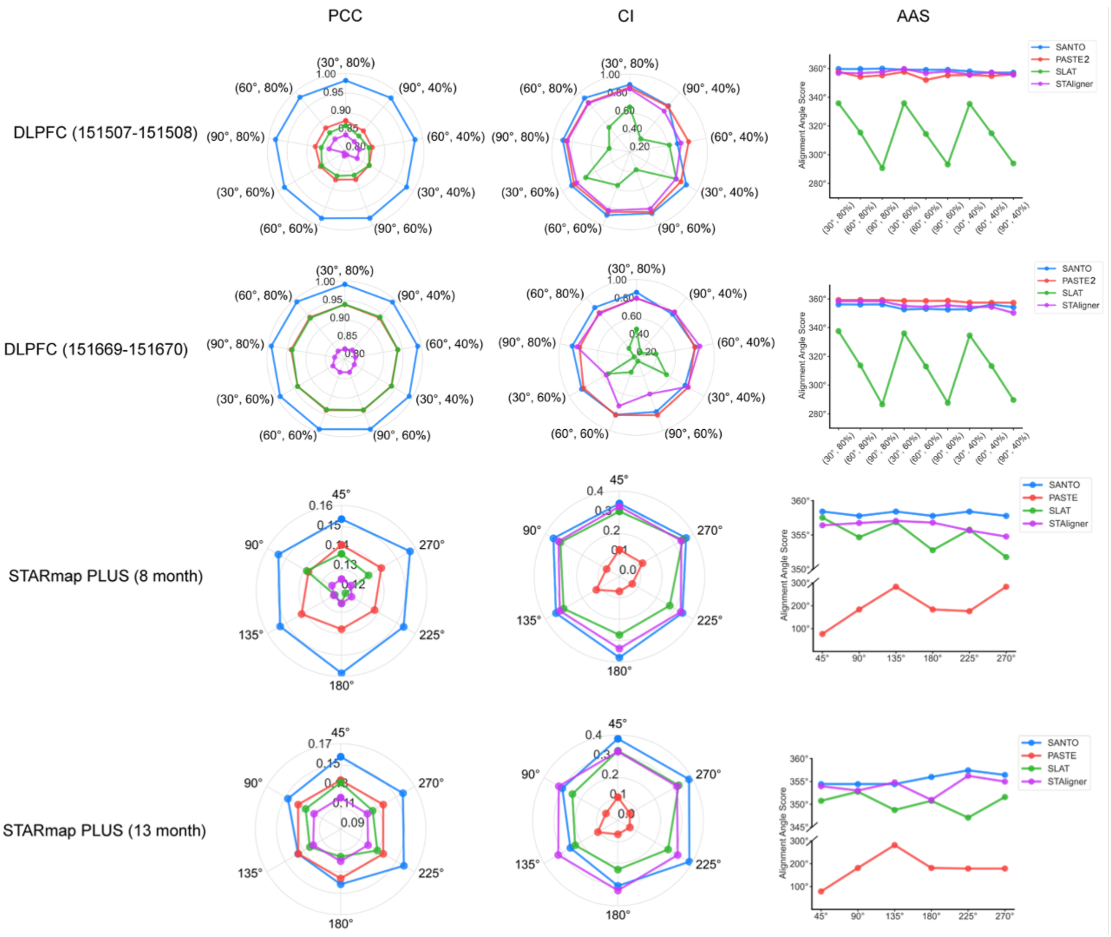

Supplementary Figure 3. PCC, CI and AAS quantifications under different conditions compared with PASTE, SLAT, STAligner and PASTE2 on the STARmap PLUS and DLPFC datasets. Source data are provided as a Source Data file.

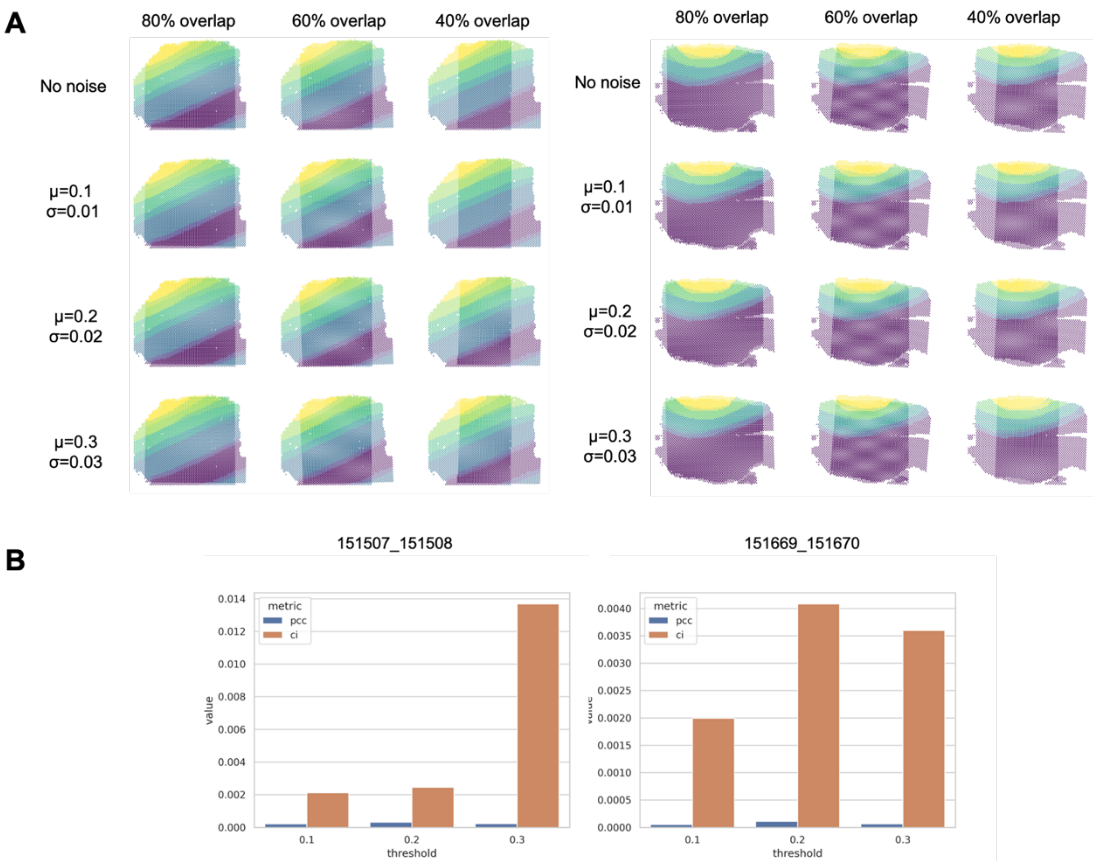

Supplementary Figure 4. A. Visualizations under different kinds of noises and overlap regions of SANTO. **B.** standard deviations of PCC and CI quantifications under different kinds of noises and overlap regions of SANTO. Source data are provided as a Source Data file.

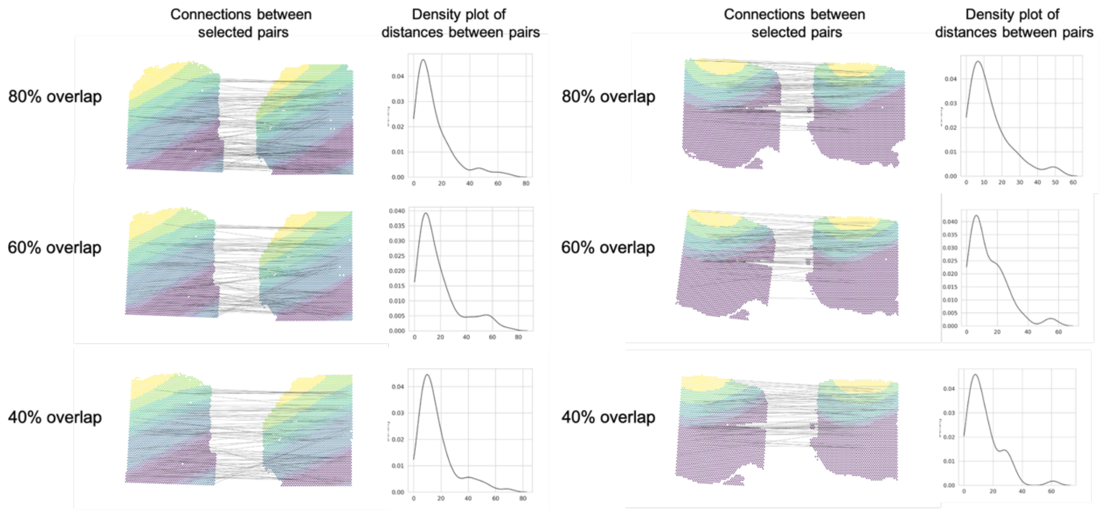

Supplementary Figure 5. Visualizations of selected pairs during coarse alignment under different percentages of overlap regions and datasets. The distributions of Euclidean distances of pairs after coarse alignment.

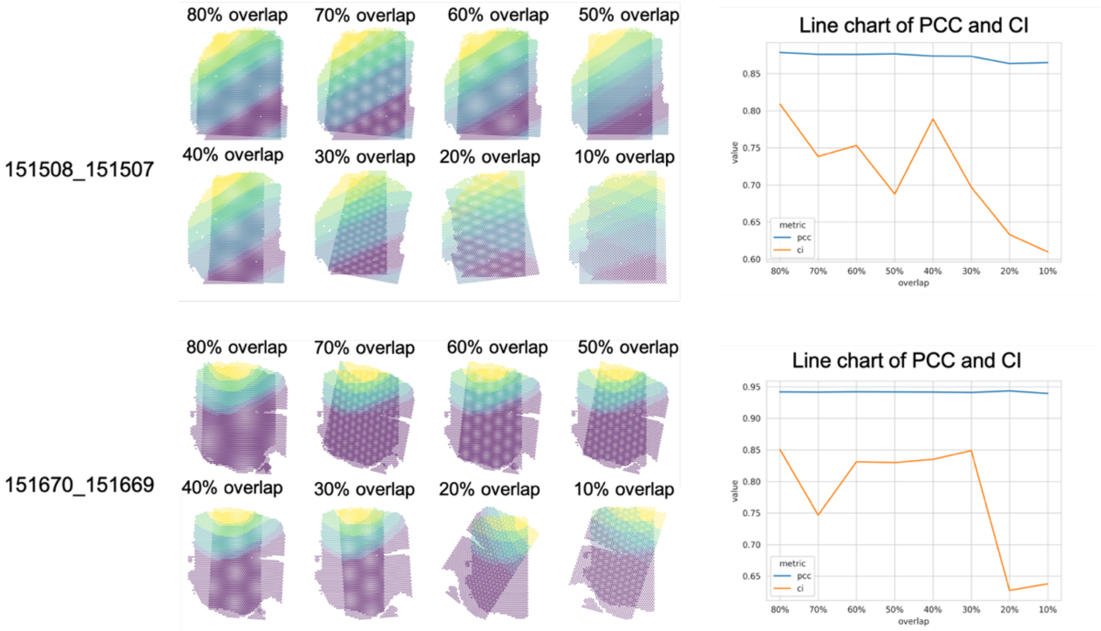

Supplementary Figure 6. Visualizations and PCC and CI quantifications of different percentages of overlap regions from 80% to 10% on the two DLPFC datasets.

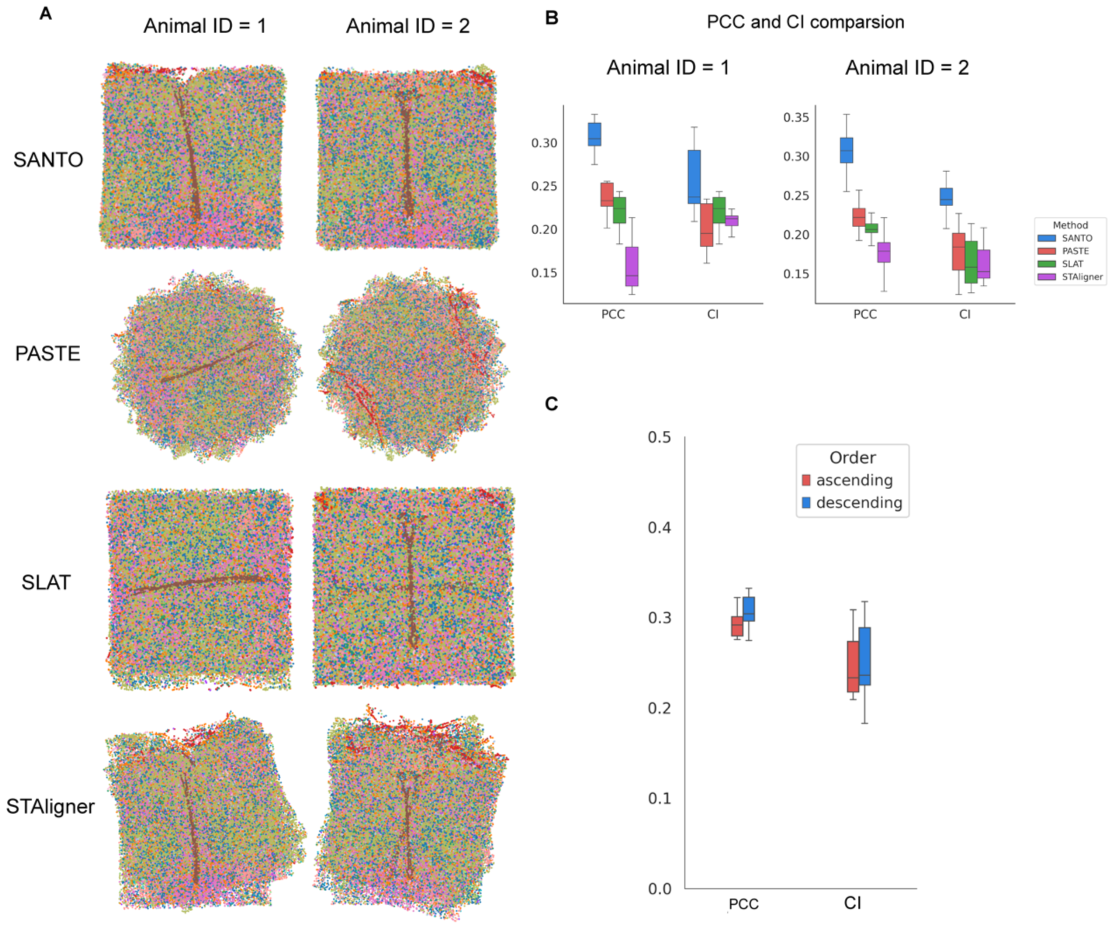

Supplementary Figure 7. A. Visualizations among SANTO, PASTE, SLAT and STAligner for two MERFISH datasets with different animal ids. **B.** PCC and CI quantifications of two MERFISH datasets among different methods. **C.** Sensitivity analysis is conducted by aligning serial slices with ascending and descending orders. The robustness of SANTO is shown by PCC and CI under two different orders. Source data are provided as a Source Data file.

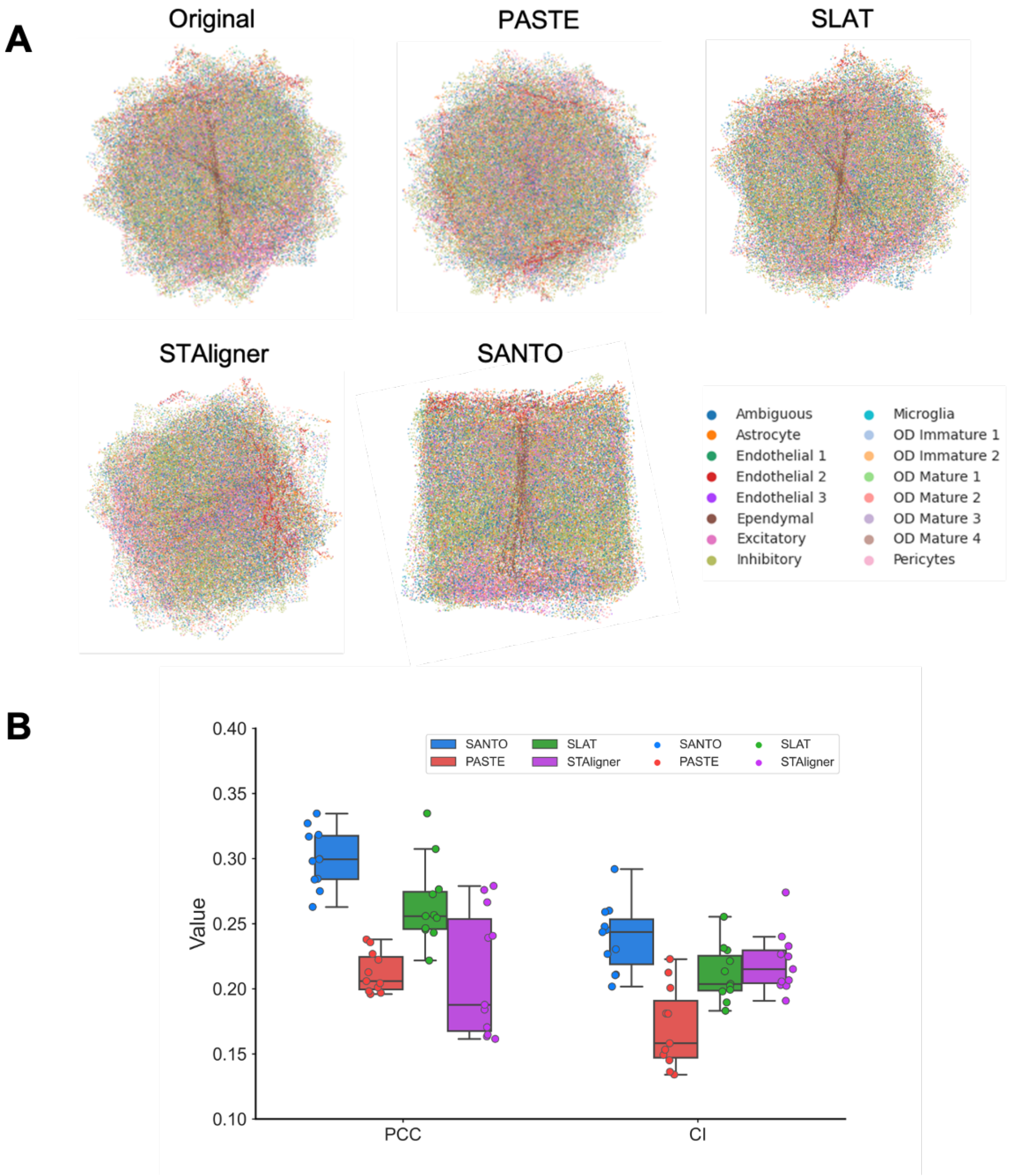

Supplementary Figure 8. A. Visualizations among SANTO, PASTE, SLAT and STAligner for MERFISH datasets (ID=2) with random rotations from 0° to 45°. **B.** PCC and CI quantifications of four methods. Source data are provided as a Source Data file.

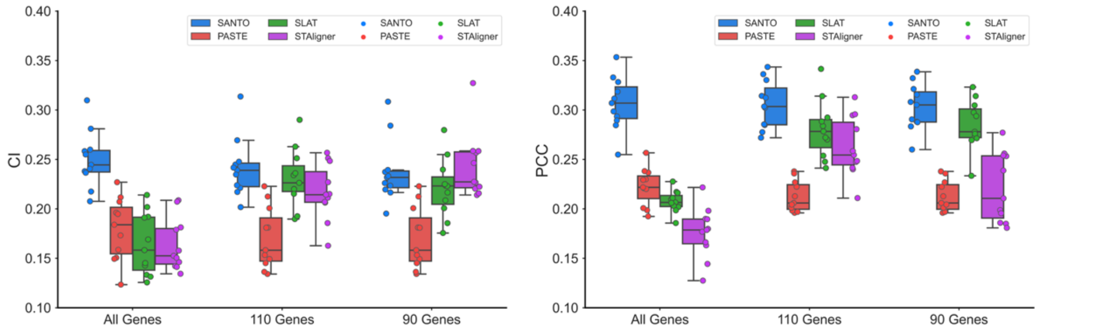

Supplementary Figure 9. PCC and CI quantifications of MERFISH datasets (ID=2) under different number of genes (all, 110 and 90 genes). Source data are provided as a Source Data file.

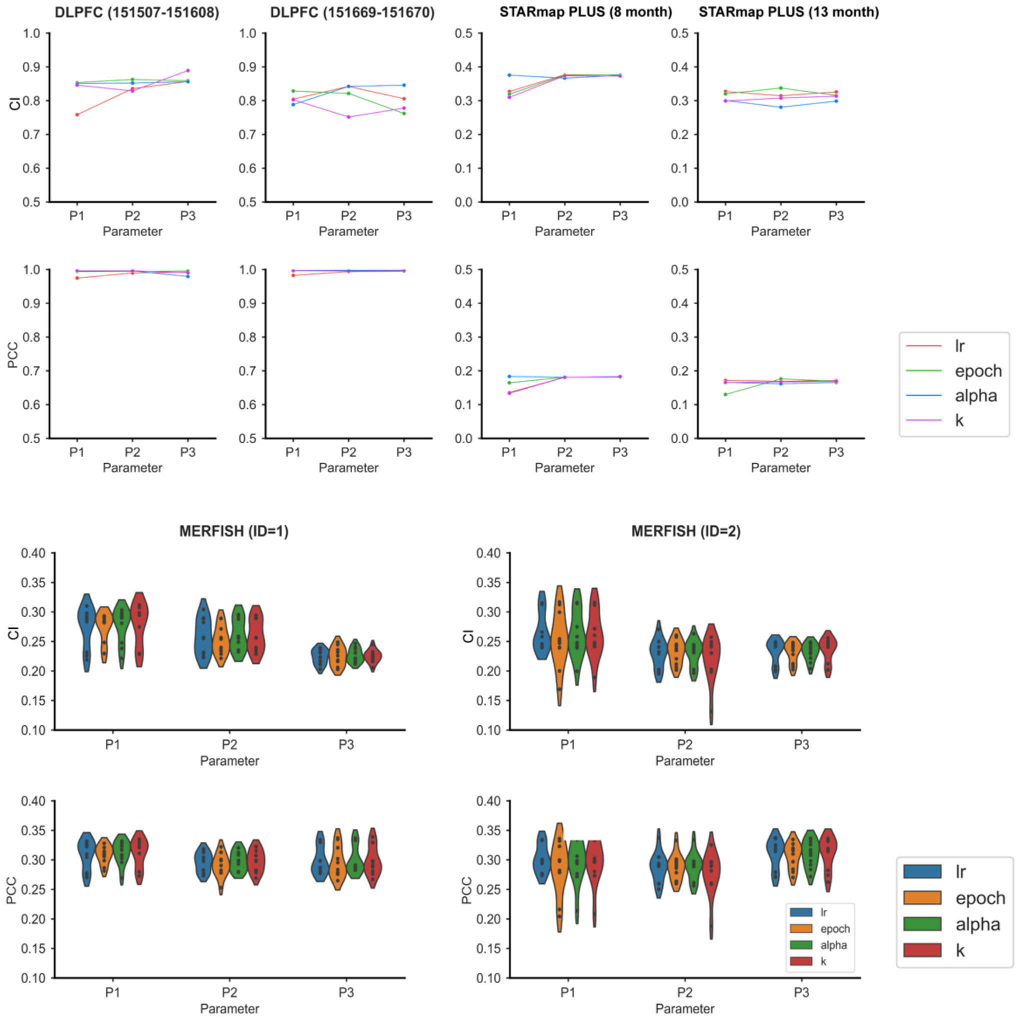

Supplementary Figure 10. Evaluation of PCC and CI on all benchmarking datasets under three different values of four hyperparameters. Source data are provided as a Source Data file.

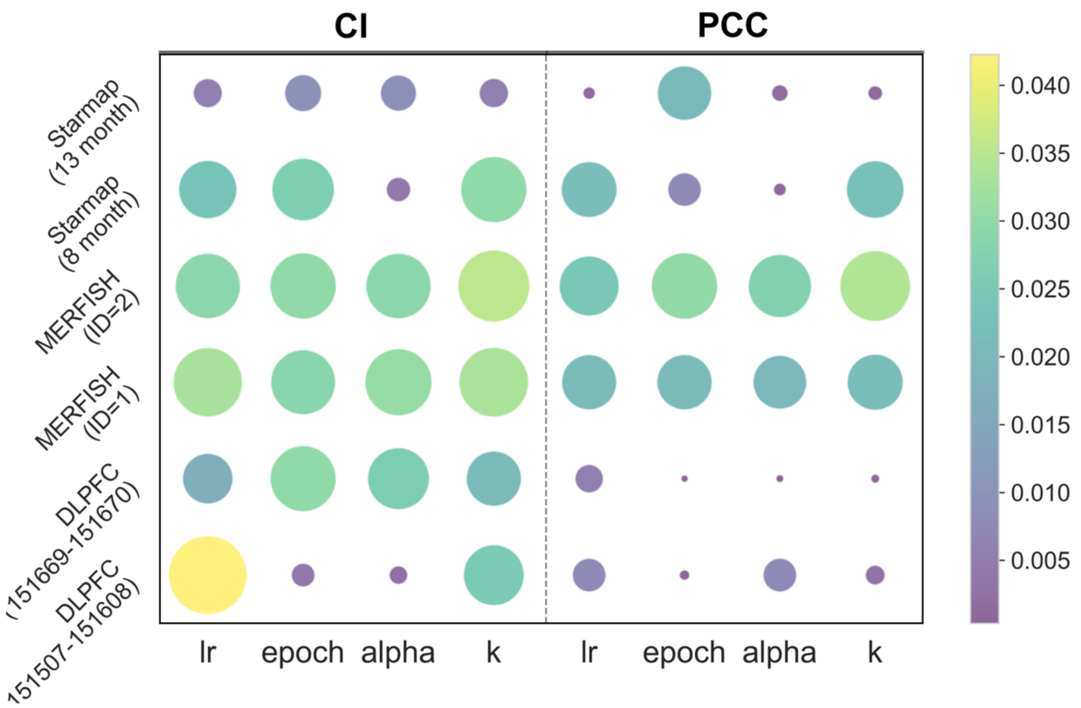

Supplementary Figure 11. Standard deviations of CI and PCC for all hyperparameters across all benchmarking datasets. Source data are provided as a Source Data file.

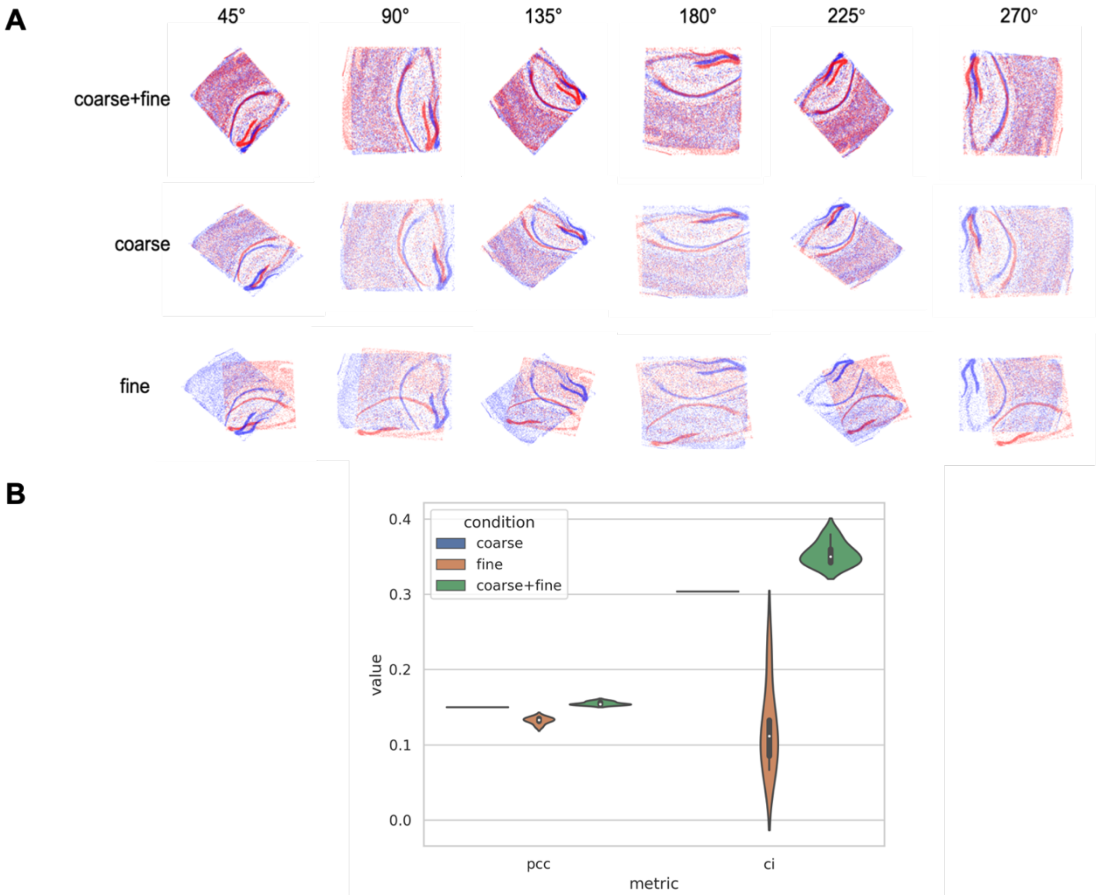

Supplementary Figure 12. A. Ablation study of coarse alignment, fine alignment and integration of coarse and fine alignment. **B.** The PCC and CI quantifications of these three conditions. Source data are provided as a Source Data file.

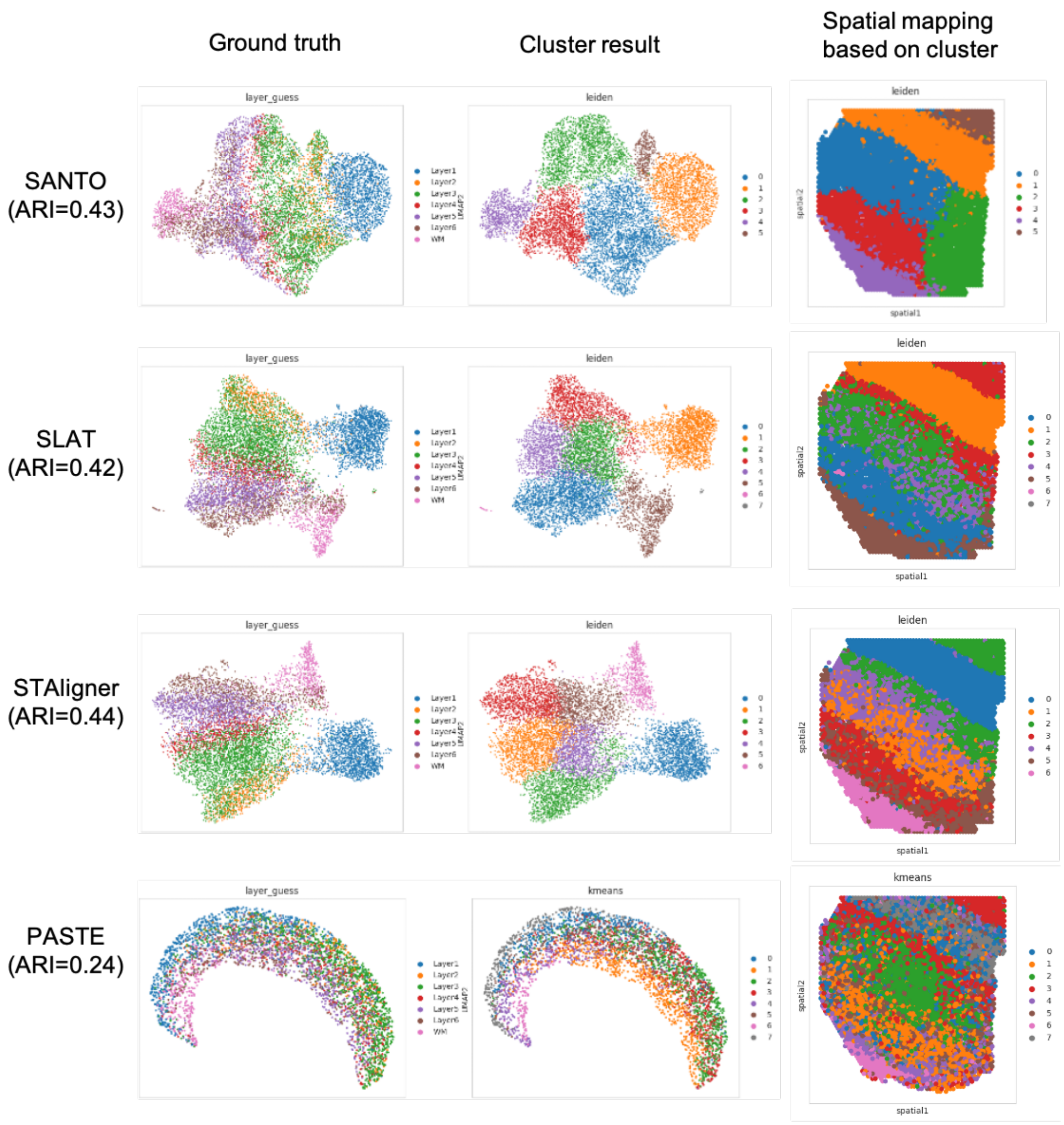

Supplementary Figure 13. Comparsion with spatial domain identification task on DLFPC dataset. For each of method, domain annotation, clustering results and the spatial mapping based on clustering results are shown. ARI score is calculated for each of them.

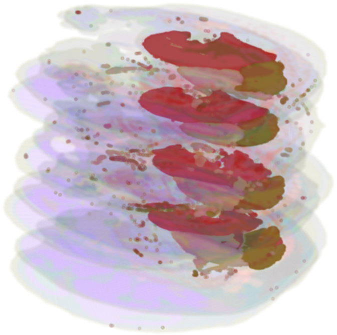

Supplementary Figure 14. Visualization of 3D-level aligned distributions of three tissues from E14.5 and E15.5, and they were spatially adjacent to each other.

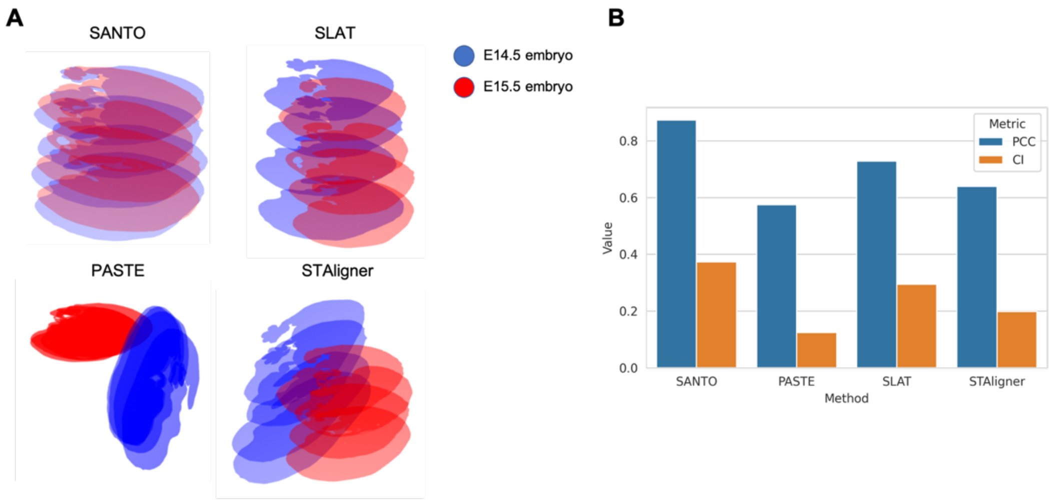

Supplementary Figure 15. A. Visualization of 3D-to-3D alignment of mouse embryos from E14.5 and E15.5 by SANTO, SLAT, PASTE and STAligner. **B.** PCC and CI quantifications of 3D-to-3D alignment of mouse embryos from E14.5 and E15.5 by SANTO, SLAT, PASTE and STAligner. Source data are provided as a Source Data file.

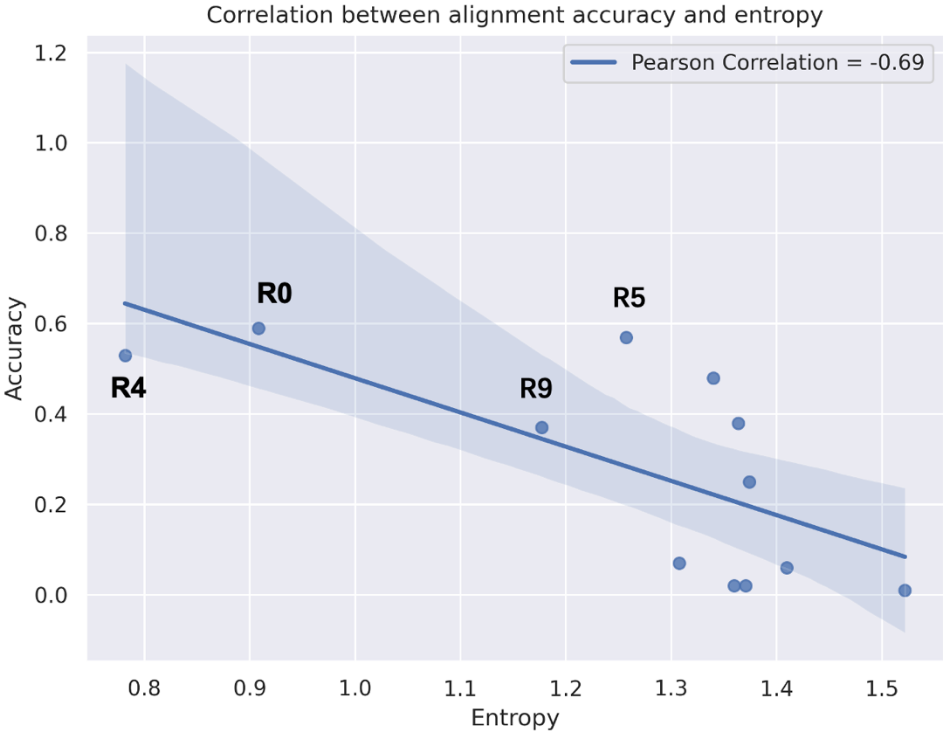

Supplementary Figure 16. Pearson Correlation between the entropy and alignment accuracy of each cell type in the alignment between RNA and H3K27ac slices from the mouse brain. The Pearson correlation is -0.69 and the p-value is 0.01252. Source data are provided as a Source Data file.

Supplementary Table 1. The calculated entropy and alignment accuracy of each cell type in the alignment between RNA and H3K27ac slices from the mouse brain.

Cell Type	Entropy	Accuracy
R4	0.781	0.53
R0	0.908	0.59
R9	1.176	0.37
R5	1.257	0.57
R8	1.307	0.07
R6	1.340	0.48
R10	1.359	0.02
R1	1.363	0.38
R11	1.370	0.02
R2	1.373	0.25
R3	1.409	0.06
R7	1.521	0.01
