# Supplementary material for: SANTO: a coarse-to-fine alignment and stitching method for spatial omics
Source: Nat Commun. 2024 Jul 18;15:6048. doi: 10.1038/s41467-024-50308-x (PMC11258319; doi:10.1038/s41467-024-50308-x)
Supplement: Supplementary file 3 — Reporting Summary [file 41467_2024_50308_MOESM3_ESM.pdf]

nature portfolio

Corresponding author(s): Xin Gao

Last updated by author(s): Jun 19, 2024

Reporting Summary

Nature Portfolio wishes to improve the reproducibility of the work that we publish. This form provides structure for consistency and transparency in reporting. For further information on Nature Portfolio policies, see our Editorial Policies and the Editorial Policy Checklist.

Statistics

For all statistical analyses, confirm that the following items are present in the figure legend, table legend, main text, or Methods section.

n/a	Confirmed	
[ ]	[x]	The exact sample size (*n*) for each experimental group/condition, given as a discrete number and unit of measurement
[x]	[ ]	A statement on whether measurements were taken from distinct samples or whether the same sample was measured repeatedly
[x]	[ ]	The statistical test(s) used AND whether they are one- or two-sided<br>*Only common tests should be described solely by name; describe more complex techniques in the Methods section.*
[x]	[ ]	A description of all covariates tested
[x]	[ ]	A description of any assumptions or corrections, such as tests of normality and adjustment for multiple comparisons
[x]	[ ]	A full description of the statistical parameters including central tendency (e.g. means) or other basic estimates (e.g. regression coefficient) AND variation (e.g. standard deviation) or associated estimates of uncertainty (e.g. confidence intervals)
[x]	[ ]	For null hypothesis testing, the test statistic (e.g. *F*, *t*, *r*) with confidence intervals, effect sizes, degrees of freedom and *P* value noted<br>*Give P values as exact values whenever suitable.*
[x]	[ ]	For Bayesian analysis, information on the choice of priors and Markov chain Monte Carlo settings
[x]	[ ]	For hierarchical and complex designs, identification of the appropriate level for tests and full reporting of outcomes
[x]	[ ]	Estimates of effect sizes (e.g. Cohen's *d*, Pearson's *r*), indicating how they were calculated

Our web collection on statistics for biologists contains articles on many of the points above.

Software and code

Policy information about availability of computer code

Data collection	No software was used for data collection since we used public available datasets.
Data analysis	The software we used were the following which were all available in github:<br>PASTE (v.1.4.0), PASTE2 (v1.0.1), SLAT (v0.2.2), STAligner (v1.0.1), and COMMOT (v0.0.3).

For manuscripts utilizing custom algorithms or software that are central to the research but not yet described in published literature, software must be made available to editors and reviewers. We strongly encourage code deposition in a community repository (e.g. GitHub). See the Nature Portfolio guidelines for submitting code & software for further information.

Data

Policy information about availability of data

All manuscripts must include a data availability statement. This statement should provide the following information, where applicable:

- Accession codes, unique identifiers, or web links for publicly available datasets
- A description of any restrictions on data availability
- For clinical datasets or third party data, please ensure that the statement adheres to our policy

STARmap PLUS: https://singlecell.broadinstitute.org/single_cell/study/SCP1375
Visium: https://doi.org/10.5281/zenodo.3689719
MERFISH: https://datadryad.org/stash/dataset/doi:10.5061/dryad.8t8s248/

Xenium and Visium (breast cancer): https://www.10xgenomics.com/products/xenium-in-situ/preview-dataset-human-breast
stereo-seq: https://db.cngb.org/stomics/mosta/
Spatial-Cut&Tag: GSE165217
MIBI-TOF: https://doi.org/10.5281/zenodo.5945388

Research involving human participants, their data, or biological material

Policy information about studies with human participants or human data. See also policy information about sex, gender (identity/presentation), and sexual orientation and race, ethnicity and racism.

Reporting on sex and gender	The sex and gender information should be clarified in the public available datasets we used.
Reporting on race, ethnicity, or other socially relevant groupings	The race, ethnicity, or other socially relevant groupings information should be clarified in the public available datasets we used.
Population characteristics	The population characteristics information should be clarified in the public available datasets we used.
Recruitment	The recruitment information should be clarified in the public available datasets we used.
Ethics oversight	The ethics oversight information should be clarified in the public available datasets we used.

Note that full information on the approval of the study protocol must also be provided in the manuscript.

Field-specific reporting

Please select the one below that is the best fit for your research. If you are not sure, read the appropriate sections before making your selection.

[x] Life sciences [ ] Behavioural & social sciences [ ] Ecological, evolutionary & environmental sciences

For a reference copy of the document with all sections, see nature.com/documents/nr-reporting-summary-flat.pdf

Life sciences study design

All studies must disclose on these points even when the disclosure is negative.

Sample size	Sample size was determined by previous published datasets which were used for our study.
Data exclusions	No experimental data was generated in our study and no data were excluded from analysis.
Replication	No replication of experimental data was performed since we did not collect any experimental data.
Randomization	Randomization of samples is not applicable in our study since we did not collect any experimental data.
Blinding	Blinding is not relevant to our study since we did not collect any experimental data.

Reporting for specific materials, systems and methods

We require information from authors about some types of materials, experimental systems and methods used in many studies. Here, indicate whether each material, system or method listed is relevant to your study. If you are not sure if a list item applies to your research, read the appropriate section before selecting a response.

Materials & experimental systems

n/a	Involved in the study	
[x]	[ ]	Antibodies
[x]	[ ]	Eukaryotic cell lines
[x]	[ ]	Palaeontology and archaeology
[x]	[ ]	Animals and other organisms
[x]	[ ]	Clinical data
[x]	[ ]	Dual use research of concern
[x]	[ ]	Plants

Methods

n/a	Involved in the study	
[x]	[ ]	ChIP-seq
[x]	[ ]	Flow cytometry
[x]	[ ]	MRI-based neuroimaging

Plants

Seed stocks	We did not use any datasets related to plants.
Novel plant genotypes	Our research is not related to plants.
Authentication	Our research is not related to plants.
